# Cell Phone Use Policies in US Middle and High Schools

**DOI:** 10.1001/jamanetworkopen.2020.5183

**Published:** 2020-05-18

**Authors:** Pooja S. Tandon, Chuan Zhou, Caroline M. Hogan, Dimitri A. Christakis

**Affiliations:** 1 Center for Child Health, Behavior, and Development, Seattle Children’s Research Institute, Seattle, Washington; 2 Department of Pediatrics, University of Washington, Seattle

## Abstract

This survey study examines cell phone policies at US middle and high schools, practices of students and faculty, and principals’ attitudes about cell phone use during school.

## Introduction

Cell phone use among middle and high school students is ubiquitous, starts at younger ages, and is negatively associated with children’s academic and social-emotional outcomes.^1^ Parents and educators are concerned about the association of cell phone use with child well-being.^2,3^ Despite these concerns, there are limited rigorous data on school cell phone use policies and practices. The aim of this study was to describe US cell phone policies and practices in middle and high schools.

## Methods

For this survey study, we obtained a listing from the National Center for Education Statistics^4^ of all US public schools serving sixth to twelfth grade in 2019. We focused on public schools because they serve more than 91% of children.^5^ The cross-sectional survey design was based on stratified probability sampling to create a nationally representative sample of middle and high schools (eAppendix in the Supplement). With use of schools listed in the sampling frame, 10% from each stratum defined by levels (middle school only, high school only, and combined schools serving grades 6-12) were randomly selected (1140 schools). Data were collected from school principals (or their representative) using an online or telephone survey. This study was approved by the Seattle Children’s institutional review board, and informed consent was waived. Data were deidentified.

The survey included questions about the presence of a cell phone policy for students and staff and about restrictions on phone use (class time, lunch time, recess, and class transitions). Additional questions related to consequences of policy violation, whether teachers used cell phones for curricular activities, and principals’ attitudes. Information about the school’s characteristics was collected. Survey items were characterized by grades served and overall. The primary outcomes were the school's cell phone use policies during times of the day. We summarized items using proportions and means (SDs) according to item type. Between-group comparisons were conducted using χ^2^ tests and analysis of variance. All tests were 2-sided, and *P* < .05 was considered statistically significant. Analyses were performed using R, version 3.6.3 (R Project for Statistical Computing).

## Results

The total sample comprised 210 schools (response rate, 18.4%) with over half the students eligible for free or reduced-priced lunch (Table 1), consistent with national estimates.^6^ Responding schools were similar to nonresponding schools in grades served, students receiving free or reduced-price lunch, and race/ethnicity except that nonresponding schools had a larger proportion of black students (16.8% vs 12.5%; *P* < .001).

**Table 1.** Characteristics of Schools

Characteristic	Median (interquartile range)		
	Middle schools (grades 6-8) (n = 106)	High schools (grades 9-12) (n = 32)	Combined schools (grades 6-12) (n = 72)
Student population	455 (280-696)	278 (194-510)	380 (170-1230)
Average class size	25 (22-29)	21 (15-25)	22 (20-25)
Staff size	28 (20-46)	25 (15-40)	31 (17-70)
Free or subsidized lunch, %	51 (25-91)	51 (26-100)	53 (29-80)
Race/ethnicity of students, %			
White	61 (11-87)	75 (39-94)	52 (25-84)
Black	3 (1-13)	2 (1-11)	4 (1-20)
Hispanic	14 (4-33)	6 (1-25)	12 (5-46)
Asian	1 (0.3-3)	1 (0-1)	2 (0.4-3)
Native American	0.5 (0-1)	0.5 (0-1.2)	0.5 (0-1)
Pacific Islander	0 (0-1)	0 (0-1)	0 (0-1)
Other	1 (0-3.5)	1 (0-1.8)	2 (0-6)
Overall graduation rate, %	NA	92 (86-97)	94 (81-98)
Students planning to attend 4 y college institutions, %	NA	37 (20-61)	45 (30-75)

Abbreviation: NA, not applicable.

A total of 103 middle schools (97%) reported having a cell phone policy for students (Table 2). Phone use during lunch and recess was not restricted by 71 middle schools (33%) and 10 high schools (69%). Across school levels, over 90% of principals supported restrictions on cell phone use for students in middle and high schools, and over 80% believed that cell phone use during school has negative consequences for social development and academics.

**Table 2.** School Policies, Practices, and Principals’ Attitudes Related to Cell Phones

Characteristic	No. (%)		
	Middle schools (grades 6-8) (n = 106)	High schools (grades 9-12) (n = 32)	Combined schools (grades 6-12) (n = 72)
Cell phone policy for students			
School has a policy regarding cell phone use by students during school time	103 (97)	29 (91)	69 (96)
Cell phone prohibited during			
Class time	89 (84)	24 (75)	56 (78)
Lunch time or recess	71 (67)	10 (31)	11 (15)
Transitions between classes	77 (73)	13 (41)	20 (28)
Other	23 (22)	3 (9)	20 (28)
Awareness of students using their cell phones during			
Class time	44 (42)	19 (59)	46 (64)
Lunch time	59 (56)	24 (75)	63 (88)
Recess	31 (29)	14 (44)	25 (35)
Transitions between classes	51 (48)	23 (72)	57 (79)
Potential consequences for a student violating the policy			
Warning	69 (65)	20 (62)	51 (71)
Cell phone taken away	98 (92)	26 (81)	61 (85)
Suspension	14 (13)	8 (25)	19 (26)
Call to parent	82 (77)	20 (62)	57 (79)
Other	25 (24)	10 (31)	17 (24)
Teachers use cell phones for curricular activities (in class)	32 (31)	9 (28)	22 (31)
Parents can communicate with their children during the day without calling their cell phone	104 (98)	30 (94)	71 (99)
Cell phone policy for staff			
School has a policy regarding cell phone use by staff during school time	49 (47)	15 (47)	35 (49)
School-level policy	22 (40)	7 (41)	15 (41)
Cell phone is prohibited during			
Class time	50 (47)	16 (50)	29 (40)
Lunch time or recess	8 (8)	1 (3)	2 (3)
Transitions between classes	20 (19)	4 (12)	3 (4)
Other	9 (8)	1 (3)	10 (14)
Principal attitudes			
There should be a cell phone use policy for students			
In middle school	100 (94)	31 (97)	66 (92)
In high school	101 (95)	30 (94)	65 (90)
Would support a policy limiting cell phone use during the school day			
Parents	97 (91)	28 (86)	63 (87)
Teachers	102 (96)	0 (100)	71 (99)
Students	51 (44)	15 (45)	31 (42)
Cell phone use during school hours has negative consequences for children’s			
Academic performance	95 (89)	26 (80)	62 (86)
Social development	93 (86)	27 (83)	61 (85)

## Discussion

This study found that although most US middle and high schools have cell phone policies in place, a notable percentage of them allow students to use their phones during class, lunch, and recess. Most principals agreed that cell phone use policies should exist at both middle and high school levels.

Whether cell phone use is occurring during class or recess, it is contributing to children’s cumulative 24-hour screen exposure. Given that most children spend the majority of their waking hours in schools, limiting phone access during the entire school day may be associated with significantly decreased exposure.

This study has several limitations, including a low response rate, although we found no differences between responding and nonresponding schools except for the proportion of black students. Also, school policies may not always be enforced and these results are subject to a response bias, wherein we may overestimate restrictive policies. Despite these limitations, to our knowledge, this was the first national survey of school cell phone policies and lays a foundation for subsequent work in this understudied area.

With increasing concerns for problematic media use, schools have a unique opportunity to create predictable screen-free time for children. Just as schools are now considered critical to helping children meet guidelines for optimal physical activity and nutrition, they should support recommendations on screen time and media use. Along with focusing on potential ramifications of cell phone use during classroom time, the consequences of students viewing screens during lunch and recess should be studied.

## Notes

Supplement. **eAppendix.** National Middle and High School Cell Phone & Related Technologies Survey
