# Supplementary material for: Cell Phone Use Policies in US Middle and High Schools
Source: JAMA Netw Open. 2020 May 18;3(5):e205183. doi: 10.1001/jamanetworkopen.2020.5183 (PMC7235688; doi:10.1001/jamanetworkopen.2020.5183)
Supplement: Supplement. — eAppendix. National Middle and High School Cell Phone & Related Technologies Survey [file jamanetwopen-3-e205183-s001.pdf]

Supplementary Online Content

Tandon PS, Zhou C, Hogan CM, Christakis DA. Cell phone use policies in US middle and high schools. *JAMA Netw Open*. 2020;3(5):e205183. doi:10.1001/jamanetworkopen.2020.5183

eFigure. National Middle and High School Cell Phone & Related Technologies Survey

This supplementary material has been provided by the authors to give readers additional information about their work.

© 2020 Tandon PS et al. *JAMA Network Open.*

eFigure. National Middle and High School Cell Phone & Related Technologies Survey

You are being asked to participate in a research study. Participation in this study is voluntary.

We will not be collecting any personal information where your participation in this study can be identified. If you agree to take part in this research study, you will be asked to complete a survey regarding your institution's policies on cell phone use in the classroom during the school day, and related technologies policies. If you have questions about the survey, or would prefer to answer via telephone, please email David Thomas at david.thomas@seattlechildrens.org

The Principal Investigator of this study is Pooja Tandon MD MPH – Seattle Children's. If you have any questions about the study you can contact Dr. Tandon at pooja.tandon@seattlechildrens.org

Please select the answer that most appropriately represents your school population

1. Grades served:

6th	7th	8th	9th	10th	11th	12th

2. What state is your school located in?
   Drop down menu of all state options

3. Is your school a charter school?
   a. Yes
   b. No

School Policy

Please select the answers that most appropriately describe your policies in regards to cell phone use, communication, and technology use in your school.

4. Is there a way for parents to communicate with their children during the day without calling their cell phone? (i.e. could they call the school office and get a message to their child)
   a. Yes
   b. No

5. Do you have a policy regarding cell phone use by staff during school time?
   a. Yes
   b. No

6. If yes, is it a district or school level policy?
   a. District
   b. School Level

7. If yes, cell phone use is prohibited for staff during (select all that apply):
   a. Class time
   b. Lunch time/Recess
   c. Transitions between classes
   d. Other? Please describe:

8. Do you have a policy regarding cell phone use by students during school time?
   a. Yes
   b. No

9. If yes, cell phone use is prohibited for students during (select all that apply):
   a. Class time
   b. Lunch time/Recess
   c. Transitions between classes
   d. Other? Please describe:

10. If yes, what are the potential consequences for a student violating the policy?
    a. Warning
    b. Cell phone taken away
    c. Suspension
    d. Call to parent
    e. Other? Please describe:

School Practices

Please select the answers that describe what you believe to be true for technology use at your school.

11. Are you aware of students using their cell phones during: (select all that apply)
    a. Class time
    b. Lunch time
    c. Recess
    d. Transitions between classes

12. Are you aware of any teachers that use cell phones for curricular activities (in class):
    a. Yes
    b. No

Attitudes

As a school administrator or principal, please share your attitudes or opinions on the following—all answers are confidential, and anonymous.

13. Do you think there should be a cell phone use policy for students in middle school?
    a. Yes
    b. No

© 2020 Tandon PS et al. *JAMA Network Open.*

14. Do you think there should be a cell phone use policy for students in high school?
    a. Yes
    b. No

15. Do you think parents would support a policy limiting cell phone use during the school day?
    a. Yes
    b. No

16. Do you think teachers would support a policy limiting cell use during the school day?
    a. Yes
    b. No

17. Do you thinks students would support a policy limiting cell phone use during: (*mark all that apply)*
    a. Class time
    b. In-between class periods
    c. During recess/lunch

18. Do you think cell phone use during school hours has negative consequences for children's academic performance?
    a. Yes
    b. No

19. Do you think cell phone use during school hours has negative consequences for children's social development?
    a. Yes
    b. No

<u>Technology Use in the Classroom</u>

Technologies in the classroom are not limited to cell phones—please respond to the following questions regarding potential laptop or tablet use during the school day.

20. Do students have access to laptops, or personal tablet devices, during the school day?
    a. Yes
    b. No

21. If yes, are laptops or tablets issued by the school, or self-purchase?
    a. Issued by school
    b. Self-purchased

22. Are students encouraged to use laptops/tablets in class for curricular activities?
    a. Yes

© 2020 Tandon PS et al. *JAMA Network Open.*

b. No

23. Are there policies in place surrounding laptop/tablet use during the school day?
    a. Yes
    b. No

Demographics:

24. What is your total numerical student population?

25. What is your average class size?

26. How many teachers do you have on staff?

27. How many students in your institution receive free or subsidized lunch?

28. What are the demographics of your student population? Please respond in percent of student body:

    - Hispanic, Mexican, or Latin American Ethnicity:
    - African-American or Black:
    - Caucasian or White:
    - Asian or Asian American:
    - Native American or Alaskan native:
    - Native Hawaiian or other Pacific Islander:
    - Other:

29. What is your overall graduation rate? (in percent who graduate)

30. Last year, what percent of your students went on to attend 4 year college institutions?

31. Are there any devices your school uses for curricular activities that we have not asked about? If so, please describe:

© 2020 Tandon PS et al. *JAMA Network Open.*

© 2020 Tandon PS et al. *JAMA Network Open.*
